# Novel Sonoguided Digital Palpation and Hydrodissection for Sural Nerve Dysfunction Mimicking Achilles Tendinopathy in a Psoriasis Patient

**DOI:** 10.3390/diagnostics15212706

**Published:** 2025-10-25

**Authors:** Yonghyun Yoon, King Hei Stanley Lam, Howon Lee, Chanwool Park, Seungbeom Kim, Minjae Lee, Jaeyoung Lee, Jihyo Hwang, Hyemi Yu, Jonghyeok Lee, Daniel Chiung-Jui Su, Teinny Suryadi, Anwar Suhaimi, Kenneth Dean Reeves

**Affiliations:** 1 Department of Orthopedic Surgery, Hallym University Gangnam Sacred Heart Hospital, Seoul 07441, Republic of Korea; lhwghm@gmail.com (H.L.); hwangjihyo36@gmail.com (J.H.); 2 IncheonTerminal Orthopedic Surgery Clinic, Incheon 21574, Republic of Korea; humanpcw94@gmail.com (C.P.); stplayer@naver.com (S.K.); mjlee951224@gmail.com (M.L.); 2wo02wo0@naver.com (J.L.); 3 International Association of Regenerative Medicine, Incheon 21574, Republic of Korea; 4 MSKUS, San Diego, CA 92084, USA; 5 The Board of Clinical Research, The International Association of Musculoskeletal Medicine, Kowloon, Hong Kong; painfreedoc22@gmail.com (T.S.); anwar@ummc.edu.my (A.S.); 6 Faculty of Medicine, The University of Hong Kong, Pokfulam, Hong Kong; 7 Faculty of Medicine, The Chinese University of Hong Kong, New Territory, Hong Kong; 8 The Board of Clinical Research, The Hong Kong Institute of Musculoskeletal Medicine, Kowloon, Hong Kong; 9 Bio Plastic Surgery Clinic, Seoul 06041, Republic of Korea; myangel315@naver.com; 10 Bareun Neurosurgery Clinic, Seoul 07273, Republic of Korea; perfectceive@gmail.com; 11 Department of Physical Medicine and Rehabilitation, Chi Mei Medical Center, Tainan 710, Taiwan; dr.daniel@gmail.com; 12 A Tempo Regeneration Center for Musicians, Tainan 700, Taiwan; 13 Department of Physical Medicine and Rehabilitation, Hermina Podomoro Hospital, North Jakarta 14350, Indonesia; 14 Department of Physical Medicine and Rehabilitation, Medistra Hospital, South Jakarta 12950, Indonesia; 15 Physical Medicine and Rehabilitation, Synergy Clinic, West Jakarta 11510, Indonesia; 16 Department of Rehabilitation Medicine, Universiti Malaya, Kuala Lumpur 50603, Malaysia; 17 Independent Researcher, Roeland Park, KS 66205, USA; deanreevesmd@gmail.com

**Keywords:** psoriasis, sural nerve dysfunction, Sonoguide Digital Palpation, ultrasound-guided hydrodissection, Achilles tendinopathy, psoriasis-associated neuropathy, neuropathic foot pain, 5% dextrose in water, crural fascia, fascial restriction

## Abstract

**Background and Clinical Significance:** Psoriasis, a chronic immune-mediated inflammatory disease, can affect musculoskeletal structures, including the Achilles tendon. Achilles pain in psoriasis patients may arise from tendinitis or neuropathic pain due to peripheral nerve dysfunction, such as sural nerve (SN) involvement, a condition frequently misdiagnosed due to limitations in conventional diagnostics. Fascial tissues are critical in nerve compression syndromes. This case explores the application of a novel quantitative Sonoguide Digital Palpation (SDP) protocol and ultrasound (US)-guided hydrodissection (HD) for SN dysfunction mimicking Achilles tendinopathy in a psoriasis patient. **Case Presentation:** A 41-year-old male with psoriasis presented with acute onset of right heel stiffness and paresthesia. Physical examination, radiographs, and ultrasound were performed. SDP, employing a validated four-criterion diagnostic framework (including fascial mobility quantification and concordant pain provocation), identified crural fascia restriction affecting SN and reproduced patient’s concordant Achilles pain. High-resolution ultrasonography provided key morphological evidence, revealing a 2.6-fold enlargement of the sural nerve’s cross-sectional area (CSA) on the affected side (13 mm^2^) compared to the asymptomatic side (5 mm^2^). Notably, a positive Tinel’s sign was elicited over the psoriatic plaque. US-guided HD was performed using 50 cc of 5% dextrose in water (D5W) without local anesthetic below the psoriatic lesion. Post-HD, the patient reported immediate and significant pain relief (Numeric Pain Rating Scale (NPRS) score reduction from 8 to 2), confirming the prompt correction of a clinically important fascial restriction, associated with improved SN mobility, objectively verified by a post-procedure SDP assessment. At 24-month follow-up, sustained symptom relief and complete functional recovery were reported. **Conclusions:** This case highlights SDP’s ability to objectively visualize and confirm fascial restriction as a cause of nerve dysfunction by quantitatively reproducing concordant pain. The objective finding of nerve swelling provides sonographic substantiation for the functional diagnosis of nerve dysfunction. This integrated diagnostic approach, combining dynamic functional assessment with morphological confirmation, offers a novel paradigm for evaluating peripheral nerve disorders. US-guided HD of the SN with D5W without local anesthetic shows promise as both a diagnostic confirmatory tool and therapeutic intervention for neuropathic Achilles pain in psoriasis patients with SN involvement, aligning with its efficacy in other peripheral neuropathies. The significant nerve swelling (13 mm^2^) provides robust morphological corroboration of the functional impairment diagnosed by SDP, offering a more comprehensive diagnostic paradigm.

## 1. Introduction

Psoriasis is a chronic, immune-mediated inflammatory disease primarily manifesting as skin lesions. It is driven by the interaction of genetic and environmental factors, leading to an aberrant immune response. Beyond dermatological involvement, psoriasis is increasingly recognized as a systemic disorder capable of affecting multiple organs, including joints, cardiovascular structures, and musculoskeletal components such as tendons and entheses [1]. Enthesitis, defined as inflammation at the insertion sites of tendons, ligaments, or joint capsules into bone, frequently affects the Achilles tendon in patients with psoriasis. Such involvement may represent an early indicator of psoriatic arthritis (PsA). According to Fiorenza et al. (2020), asymptomatic enthesitis at the Achilles tendon insertion site is detected via ultrasound in approximately 30–50% of psoriasis patients, which might later progress to clinical symptoms [2].

When psoriasis patients experience Achilles tendon-related pain, potential causes include tendinitis and neuropathic pain from peripheral nerve dysfunction. Fascial tissues are critical in nerve compression syndromes, acting as dynamic anatomical constraints whose mobility impairments directly contribute to neural compression and pain generation [3]. The crural fascia, a deep fascial layer of the lower leg, surrounds the calf muscles and forms compartments through which peripheral nerves travel with the fascia, e.g., the sural nerve in the fascia between the medial and lateral gastrocnemius [4,5,6]. Its primary function is to provide structural support and facilitate muscle action while permitting physiologic nerve gliding during movement. Diagnosing peripheral nerve dysfunction in psoriasis patients can be challenging due to overlapping symptoms with other musculoskeletal disorders and the limitations of traditional imaging and electrophysiological techniques. Traditional methods like X-rays primarily visualize bony structures and are unable to depict soft tissue abnormalities such as fascial thickening, adhesions, or subtle nerve compressions [7]. MRI can visualize soft tissues, but its resolution may be insufficient to detect subtle fascial restrictions or early-stage nerve pathology, and it often lacks the dynamic assessment capabilities needed to evaluate nerve mobility during movement [8]. Electrodiagnostic testing (EDX) demonstrates poor sensitivity for functional neurological conditions due to its inability to assess small-fiber pathology, with sural sensory nerve action potentials showing only 66% sensitivity for axonal polyneuropathy and even poorer performance for isolated demyelination [9,10,11,12]. Diagnostic nerve blocks—while commonly used—suffer from unreliable predictive value for surgical outcomes. Recent evidence reveals area-under-curve (AUC) metrics as low as 0.35 (95% CI: 0.077–0.62) for lidocaine blocks, indicating performance worse than random chance (AUC 0.5), compounded by 29% false-positive rates from placebo effects and significant false-negative outcomes [13]. These limitations underscore the need for techniques that directly visualize fascial–neural interfaces while dynamically quantifying restrictive pathology—a gap addressed by novel diagnostic protocols integrating layer palpation with ultrasound [3].

The DANBIO study, a Danish registry for biological treatment in rheumatology—a clinical quality assurance database initiated in 2000—reported a notably higher prevalence of neuropathic pain among PsA patients compared to those with rheumatoid arthritis (RA) or other spondyloarthritis (SpA) [14]. This study utilized the PainDETECT questionnaire (PDQ) to evaluate neuropathic pain components, finding a notably higher prevalence of neuropathic pain features in PsA patients (28%) compared to those with RA (17%) and axial SpA (13%), suggesting neuropathic pain as a clinically significant issue in PsA [15,16]. However, the clinical or imaging parameters of neuropathic pain in PsA patients remain underexplored, and the specific mechanisms underlying this increased neuropathic pain burden are not well understood. Given this, it is important to recognize that chronic foot and ankle pain—frequently reported among patients with musculoskeletal or systemic inflammatory conditions—may arise from diverse etiologies, including pathologies of ligaments, tendons, nerves, vascular structures, or skin. Among these, nerve compression syndromes such as sural nerve dysfunction, although relatively uncommon, should not be overlooked as potential contributors to persistent heel or lateral foot pain [4,17,18,19,20]. Traditional diagnostic methods, such as physical examination and standard radiography, often lack the sensitivity to detect subtle fascial abnormalities contributing to nerve pathology. Emerging techniques like Sonoguided Digital Palpation (SDP) address this gap by combining real-time ultrasound with quantitative fascial mobility assessment to localize restriction sites and reproduce concordant pain—a validated approach in other peripheral neuropathies [3]. Crucially, high-resolution ultrasound can also provide objective morphological evidence, such as nerve swelling quantified by cross-sectional area (CSA) measurement, to substantiate the functional diagnosis of entrapment [21,22,23].

US-guided HD has emerged as an effective treatment for persistent foot pain, particularly in cases resistant to conservative treatment. This technique involves the injection of fluid around nerves to physically separate them from adhesions, thereby relieving pressure on nervi nervorum and vasa nervorum, ultimately alleviating pain and improving function [24]. When performed with 5% dextrose in water (D5W) without local anesthetic, HD serves dual diagnostic and therapeutic roles: immediate functional recovery confirms reversible neural dysfunction, while mechanical adhesiolysis combined with dextrose-mediated anti-neuroinflammatory effects sustains relief [25]. Recent case reports indicate that sural nerve dysfunction, unresponsive to conservative treatment, can be safely and effectively managed through US-guided HD [17,26,27], a finding consistent with positive outcomes observed in other peripheral nerve conditions such as carpal tunnel syndrome [17,27,28]. Additionally, high-volume image-guided injection (HVIGI), which targets increased neovascularization and nerve infiltration in chronic Achilles tendinopathy, has demonstrated significant clinical efficacy [29,30,31].

Based on these findings, and building upon validated protocols for fascial restriction diagnosis and management, we report a case of sural nerve dysfunction mimicking Achilles tendinopathy in a patient with psoriasis. This case was uniquely diagnosed using a combination of SDP and definitive sonographic evidence of nerve enlargement (2.6-fold increase in cross-sectional area) and subsequently managed with US-guided HD with D5W without local anesthetic performed below the psoriatic lesion.

## 2. Case Presentation

A 41-year-old Asian male warehouse worker presented with acute exacerbation of right heel pain and paresthesia, resulting in severe ambulatory impairment requiring an antalgic gait. His symptoms began two to three months prior as intermittent mild stiffness and paresthesia, which persisted despite thrice-weekly physiotherapy and regular nonsteroidal anti-inflammatory drug (NSAID) therapy. Within 48 h prior to presentation, he experienced rapid symptom progression characterized by debilitating pain (Numeric Pain Rating Scale [NPRS] 8/10), profound stiffness, and muscle tightness that substantially compromised occupational duties and activities of daily living. The patient denied prior similar episodes or recent traumatic incidents.

**Figure 1:**
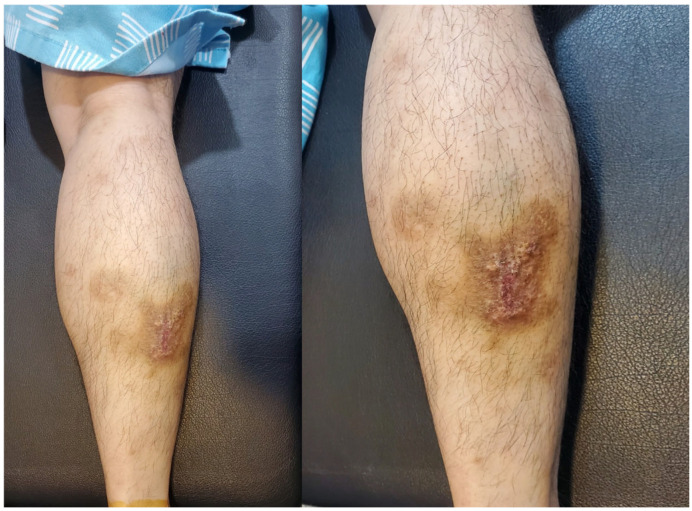
Psoriatic skin lesion on the right calf of the presented patient.

**Figure 2:**
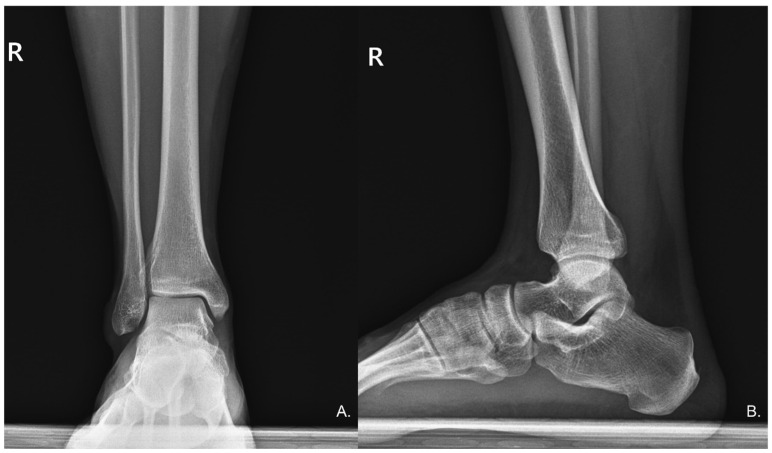
(**A**) Anteroposterior (AP) and (**B**) Lateral radiographs of the right ankle show no radiographic evidence of deformities or other significant bony abnormalities.

Physical examination revealed an antalgic gait during ambulation to the consultation area. Palpation elicited significant tenderness in the posterior calcaneal region, with passive dorsiflexion of the right ankle provoking substantial discomfort. A well-demarcated psoriatic plaque was observed on the right calf (Figure 1), correlating with the patient’s six-year history of psoriasis for which no active treatment was being administered. Neuromuscular assessment demonstrated decreased pin-prick sensation in the sural nerve distribution distal to the psoriatic plaque. A Tinel’s sign was positive, with percussion over the psoriatic plaque eliciting concordant paresthesias radiating distally along the anticipating pathway of the sural nerve. The remainder of the comprehensive neurological evaluation of the lower limbs yielded unremarkable findings. Standard anteroposterior and lateral radiographs of the ankle revealed no structural abnormalities (Figure 2).

### 2.1. Diagnosis of Sural Nerve Involvement Using Sonoguide Digital Palpation

**Figure 3:**
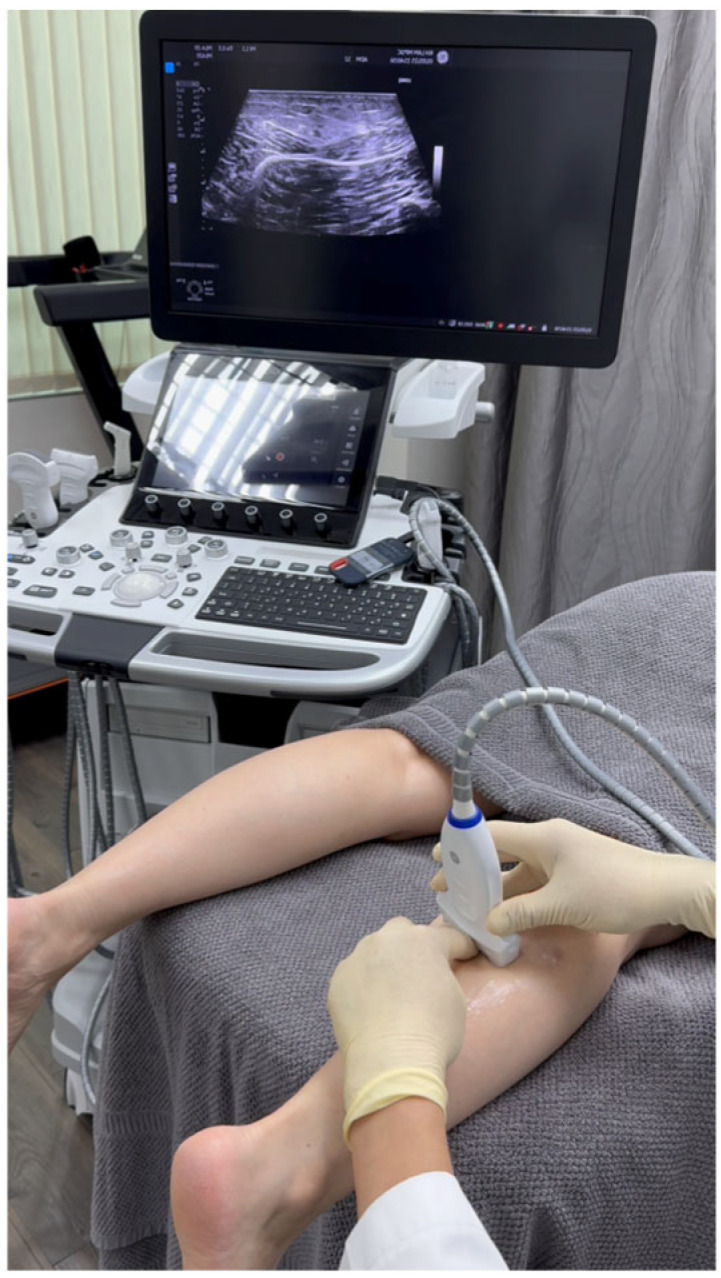
Setup for Sonoguide Digital Palpation (SDP) to assess sural nerve (SN). The palpating finger is positioned centrally on the ultrasound transducer to accurately correlate palpation with real-time imaging. Rhythmic palpation is applied in both lateral-to-medial and medial-to-lateral directions over the suspected SN location within the fatty triangle of the crural fascia, between the medial and lateral gastrocnemius muscles. The mobility of the SN and the lesser saphenous vein relative to the surrounding soft tissues, along with the patient’s pain response, are carefully evaluated during SDP. Note the palpating finger of the physician is pointing to the approximate location of the sural nerve (SN) and lesser saphenous vein (LSV).

Sonoguide Digital Palpation was employed to diagnose and visualize the suspected sural nerve dysfunction (Figure 3 and Video S1).

**Figure 4:**
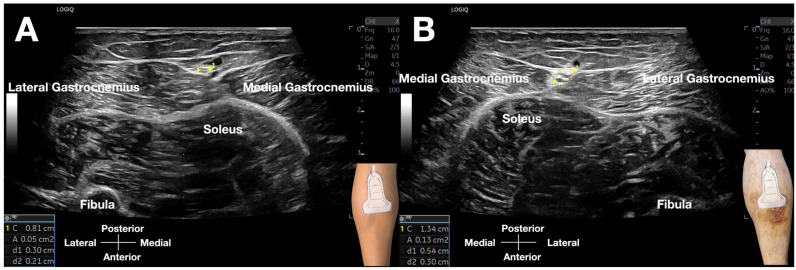
Comparative cross-sectional ultrasound images of the sural nerve (SN). (**A**) Asymptomatic left leg: The SN (calipers) within the crural fascia’s fatty triangle demonstrates a normal cross-sectional area of 5 mm^2^, with preserved fascicular architecture and no perineural edema. (**B**) Symptomatic right leg: The SN (calipers) is significantly enlarged, with a cross-sectional area of 13 mm^2^, loss of fascicular definition, and surrounding hypoechoic edema, consistent with nerve pathology and fascial restriction.

Sonoguided Digital Palpation (SDP) represents a dynamic diagnostic technique integrating real-time ultrasonography with targeted layer palpation [3], grounded in established osteopathic principles of myofascial and visceral assessment [32,33,34,35,36,37,38]. This methodology enables direct visualization and quantification of tissue tension abnormalities and interfacial friction dynamics. In the present case, SDP was employed to evaluate the crural fascia (CF), wherein restricted gliding mobility relative to superficial adipose tissue or underlying gastrocnemius musculature signified fascial pathology (Figure 4 and Video S1). A critical advantage of this approach is the capacity for real-time contralateral comparison, providing an internal control for biomechanical assessment.

The technique was executed with controlled digital pressure (estimated 2–4 kg/cm^2^) applied rhythmically for 10–15 s intervals at each assessment site, utilizing alternating lateral-to-medial and medial-to-lateral vectors (Figure 3, Video S1). Diagnostic interpretation followed a validated four-criterion protocol [3]: (1) Quantitative fascial mobility restriction (≤5 mm transverse excursion versus >5 mm contralaterally); (2) Precise reproduction of concordant Achilles pain upon sural nerve (SN) provocation; (3) Identification of crural fascial gliding impairment; (4) Sonographic evidence of significant nerve swelling, with a cross-sectional area (CSA) of 13 mm^2^ on the affected side, compared to 5 mm^2^ on the contralateral, asymptomatic side (Figure 4, Video S2), alongside perineural edema and fibrotic tissue changes.

Selective application of SDP within the crural fascia’s fatty triangle—an anatomical space bounded by the medial and lateral gastrocnemius muscles—facilitated assessment of sural nerve (SN) and lesser saphenous vein (LSV) mobility relative to adjacent structures. Diagnostic criteria for symptomatic SN involvement included: reproduction of characteristic Achilles pain upon neural palpation, palpable tethering with restricted nerve mobility, and increased gliding resistance. Additionally, layered palpation excluded myofascial trigger points in the gastrocnemius and soleus muscles through depth-specific pressure application; the absence of twitch responses further differentiated neuropathic from myogenic pain etiologies.

### 2.2. Ultrasound-Guided Hydrodissection for Sural Nerve Dysfunction

Given the patient’s refractory symptoms despite conservative management and SDP-confirmed sural nerve dysfunction, combined with his acute severe pain presentation and preference to avoid electrodiagnostic delays, ultrasound-guided hydrodissection using 5% dextrose in water (D5W) without local anesthetic was selected as a combined diagnostic and therapeutic intervention [25]. This approach aligns with established protocols for managing acute compressive neuropathies where immediate functional response confirms treatable pathology [25]. Informed consent was obtained following detailed discussion of risks and benefits.

**Figure 5:**
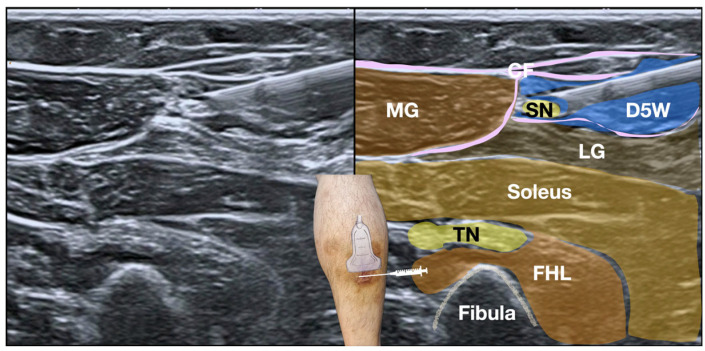
Ultrasound image of the lateral calf region demonstrating in-plane needle approach for hydrodissection of the sural nerve. Note the hyperechoic needle tip is visualized advancing toward the nerve for targeted injectate delivery via a lateral-to-medial approach. D5W, 5% dextrose in water; FHL, flexor hallucis longus; LG, lateral gastrocnemius; MG, medial gastrocnemius; SN, sural nerve; TN, tibial nerve.

**Procedure:** Technical execution adhered to systematic fascial release methodology [3]: A 24-MHz linear high-frequency transducer (Canon i24LX8, Canon Medical Systems, Tustin, CA, USA) was utilized with strict sterile technique involving 2% chlorhexidine in 75% alcohol skin preparation and sterile probe covering. Superficial cutaneous anesthesia with 1% lidocaine preceded the procedure to minimize discomfort without confounding neural feedback. Using a 25-gauge × 2-inch hypodermic needle, 50 mL of D5W without adjuvant agents was administered as the hydrodissection solution (Figure 5, Video S3).

The hydrodissection technique was systematically applied [3]: Initial hydraulic dissection commenced with the injectate creating a tissue-separating halo anterior to the needle tip. Initial circumferential hydrodissection targeted the SN’s inferior aspect (needle bevel superiorly oriented), followed by superior aspect hydrodissection (bevel inferiorly oriented) to achieve 360° perineural fluid distribution. Primary intervention focused on the SDP-identified maximum tenderness site—characterized by concordant pain reproduction and sonographic evidence of fibrotic tethering within the crural fascia.

The ‘fascial unzipping’ technique, as previously described [3], was employed; the proximal and distal SN segments were subsequently addressed through transducer-needle pivoting from a single-entry point, enabling release along >5 cm of the SN course. Procedural endpoints included: (1) Sonographic confirmation of a circumferential fluid halo (>1 mm surrounding SN); (2) Immediate intraprocedural NPRS reduction to ≤3 (specifically to 2 in this case); (3) Restored nerve mobility on dynamic assessment.

SDP had confirmed sural nerve involvement through precise reproduction of concordant pain. Post-hydrodissection, real-time ultrasonography documented complete perineural fluid distribution surrounding the SN within the crural fascia’s fatty triangle, indicating successful adhesiolysis (Video S3). The patient reported immediate functional improvement with the NPRS reduction from 8 to 2 during ambulation and complete resolution of Achilles stiffness. Post-procedural SDP objectively verified therapeutic efficacy through two parameters: (1) Absence of pain provocation at the previously symptomatic site; (2) Restored nerve mobility > 5 mm during dynamic assessment—quantitatively confirming reversal of functional impairment [3,25]. Ultrasound visualization demonstrated transition from pre-procedural tethering to normal fluid gliding dynamics, correlating with clinical recovery (Figure 5, Video S3).

The hydrodissection procedure was performed in a single therapeutic session with no additional interventions required during the 24-month follow-up period. The intervention achieved sustained therapeutic outcomes: At 24-month follow-up, telephone interview confirmed complete symptom resolution and functional restoration without recurrence. This durability aligns with long-term efficacy patterns observed in fascial restriction neuropathies managed through analogous protocols [3].

## 3. Discussion

This case extends the diagnostic–therapeutic paradigm established for fascial restriction neuropathies [3,25] to include psoriasis-related sural nerve dysfunction. Beyond demonstrating complete resolution of neuropathic heel pain through ultrasound-guided HD, this report provides a critical advancement in diagnostics: the integration of dynamic functional assessment (SDP) with objective morphological evidence (a 2.6-fold increase in nerve cross-sectional area). Our approach leveraged dual mechanisms: mechanical adhesiolysis via ‘fascial unzipping’ and D5W-mediated anti-neuroinflammatory effects. These protocols have been validated for long thoracic and acute radial neuropathies [3,25]. While high-volume image-guided tendon injection effectively addresses tendinopathy-related neovascularization [21,29], our intervention highlights a distinct therapeutic mechanism that involves both mechanical and biochemical effects of HD around the proximal sural nerve.

**Figure 6:**
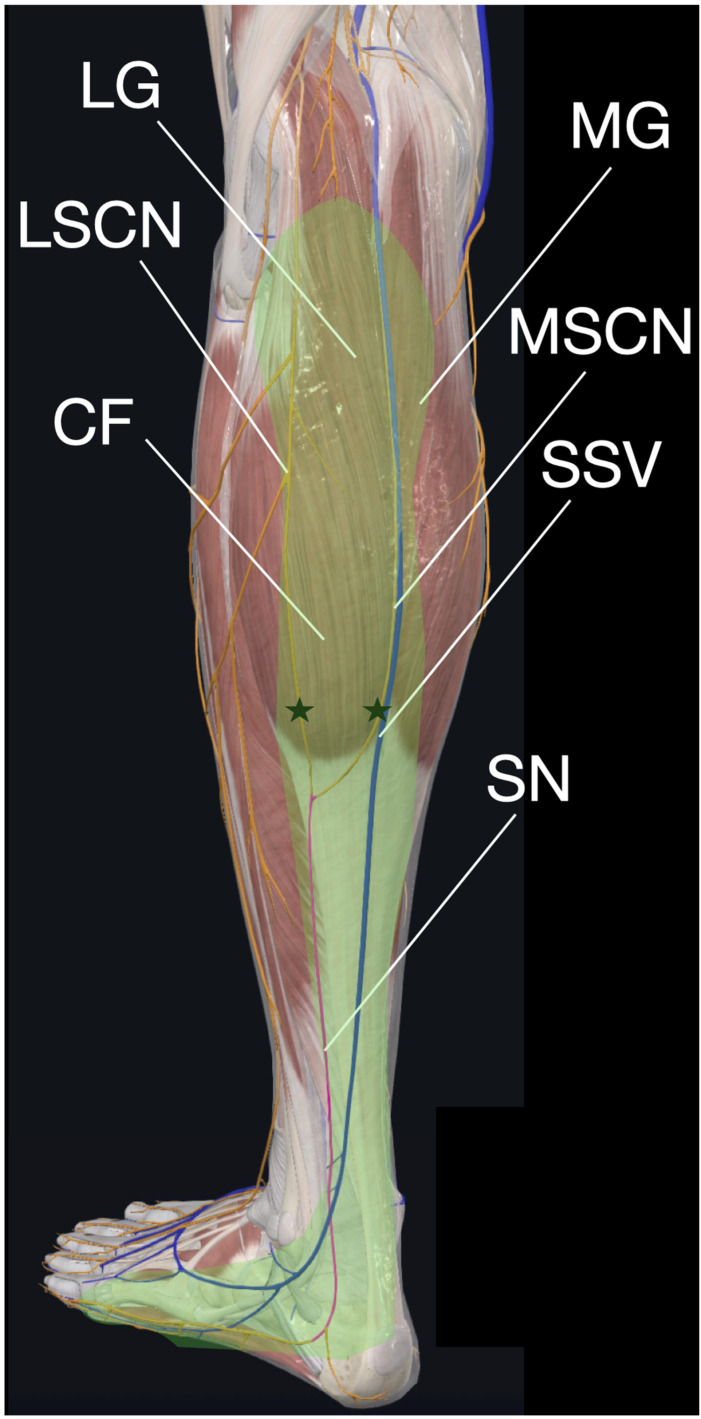
Anatomical course of the sensory sural nerve (SN) showing: Contributions from medial (MSCN) and lateral (LSCN) sural cutaneous nerves; Relationship with medial and lateral gastrocnemius heads (MG/LG), crural fascia (CF) and small saphenous vein (SSV); Transition from deep fascial course to subcutaneous path near Achilles myotendinous junction (★); Sensory distribution to posterolateral leg/lateral foot (shaded region).

Anatomically, the sural nerve (SN) is a purely sensory nerve formed by contributions from the tibial nerve (medial sural cutaneous nerve) and common peroneal nerve (lateral sural cutaneous nerve) [4,5,6,18,19]. As illustrated in Figure 6, the SN courses distally within the crural fascia between the medial and lateral gastrocnemius muscles before piercing the fascia to become subcutaneous near the myotendinous junction [4,5,6,18,19]. Its exclusive sensory innervation of the posterolateral distal calf, lateral ankle, and dorsolateral foot [5,19] explains why dysfunction produces neuropathic symptoms, such as pain and paresthesia, without motor deficits. This is consistent with our patient’s presentation of heel stiffness and paresthesia in the absence of muscle weakness.

Sural neuropathy may contribute to balance impairment through three mechanisms: (i) disrupted somatosensory feedback that impairs proprioception [22], (ii) pain-induced antalgic gait adaptations [23], and (iii) fascial adhesions that restrict ankle mobility [39]. The resolution of balance deficits post-hydrodissection supports a neuropathic etiology rather than primary tendinopathy.

This case report presents several novel contributions to the existing literature on sural nerve pathology. Foremost, it provides a detailed account of sural nerve dysfunction occurring in direct anatomical proximity to a psoriatic skin lesion. We provide objective sonographic evidence of substantial nerve enlargement, with a 2.6-fold increase in cross-sectional area (13 mm^2^ vs. 5 mm^2^ contralaterally), offering morphological confirmation of nerve pathology underlying the functional impairment. This spatial relationship suggests a potential link between localized psoriatic inflammation and nerve compression, thereby expanding our understanding of psoriasis-related musculoskeletal complications beyond classical enthesopathy and arthritis [1,2]. Second, the application of SDP constitutes an innovative diagnostic advancement [3]. By integrating dynamic ultrasound imaging [36,40] with layer-specific palpation [32,33,34,35], SDP enabled direct visualization of fascial restriction as the primary etiology while reproducibly eliciting the patient’s concordant pain. The combination of functional assessment through SDP with morphological evidence of nerve swelling provides a comprehensive diagnostic approach that addresses limitations of conventional techniques. This addresses critical limitations of static imaging modalities in detecting subtle fascial pathology [7,8,32,33,34,35]. Third, our findings establish fascial pathology, specifically crural fibrosis and adhesion in a psoriatic patient, as a central mechanistic driver of nerve dysfunction in this context [4,41,42,43], distinguishing this case from prior reports of idiopathic or post-traumatic sural neuropathy [17,26,27]. While existing literature supports hydrodissection for sural nerve release [17,26,27], the integration of SDP for diagnosis and the focus on fascial etiology within a psoriasis-specific framework represent significant clinical and pathophysiological advancements.

The efficacy of US-guided HD for sural nerve dysfunction—though relatively understudied—is supported by emerging evidence from case reports and small studies. Fader et al. (2015) utilized ultrasound imaging to identify sural nerve entrapment characterized by edema and scar formation, which was successfully treated via percutaneous hydrodissection [27]. Similarly, Tople (2021) and Omodani (2023) reported effective pain relief after hydrodissection for localized sural neuropathy [17,26]. Tople and Bhuyan (2021) documented complete pain resolution following the percutaneous ultrasound-guided hydrodissection of the sural nerve [17]. This intervention mechanically separates nerves from surrounding constrictive tissues, potentially relieving mechanical compression and associated neuropathic symptoms [44]. The surrounding fascia, when thickened or adhered, can directly contribute to nerve compression, emphasizing the importance of addressing fascial pathology in these cases. Tople and Bhuyan (2021) [17] demonstrated substantial and sustained pain relief in patients with refractory sural nerve entrapment following US-guided HD, noting no recurrence. Lam et al. (2020) [24] proposed a mechanism involving mechanical separation of perineural adhesions, alleviating compression of the nervi nervorum and vasa nervorum—structures crucial for neural innervation and vascular supply. These reports underscore ultrasound’s role in visualizing entrapment sites (e.g., edema/scar tissue) and guiding precise fluid injection to release nerves. In addition to these mechanical effects, the injected 5% dextrose solution may exert biochemical effects, such as alleviation of perineural glycopenia or modulation of pain via downregulation of nociceptive ion channels, including transient receptor potential vanilloid receptor-1 (TRPV1) [28,45,46] and anti-neurogenic inflammation effects [47,48,49]. We suggest that both mechanical and biochemical mechanisms may have contributed to the clinical improvement observed in our patient.

### 3.1. Neuropathic Pain in Psoriatic Arthritis: An Unexplored Mechanistic Pathway

The DANBIO registry findings, which documented a significantly higher prevalence of neuropathic pain in PsA patients (28%) compared to RA (17%) and axial SpA (13%), underscore the clinical importance of recognizing neuropathic pain as a feature of psoriatic disease. However, while the DANBIO study quantified the prevalence of neuropathic pain, it did not elucidate the specific mechanisms underlying this phenomenon. Our case presents an unusual and previously undocumented mechanistic pathway: direct anatomical compression of the sural nerve by localized psoriatic inflammation. The spatial relationship between the psoriatic plaque and the site of sural nerve dysfunction in our patient is particularly noteworthy. Unlike the more commonly described systemic or joint-related causes of neuropathic pain in PsA, our patient’s nerve dysfunction appeared to be directly related to the local inflammatory milieu created by the psoriatic skin lesion. This mechanistic distinction is critical: while systemic inflammation in PsA may predispose to generalized peripheral neuropathy or nerve compression at multiple sites, our case demonstrates a focal, topographically related pathway wherein a localized psoriatic lesion creates a microenvironment of inflammation sufficient to compress and inflame an adjacent peripheral nerve. The proposed mechanism—localized psoriatic inflammation triggering fascial fibrosis and subsequent nerve compression—represents a previously unexplored pathway that warrants further investigation in larger cohorts.

### 3.2. Diagnostic Paradigm Shift: Moving Beyond Traditional Limitations

Our approach offers a complementary pathway to conventional diagnostic algorithms. In this case, the diagnosis was supported by the classic clinical finding of a positive Tinel’s sign at the site of maximal tenderness, which helped to localize the source of the neural irritation. SDP [3] added a crucial dynamic and quantitative layer to this diagnosis by providing real-time visualization of fascial dynamics and restricted nerve mobility responsible for this positive sign. The technique’s ability to reproduce concordant pain during fascial assessment provides functional validation absent in static imaging. Similarly, our D5W hydrodissection protocol fundamentally differs from anesthetic blocks by mechanically disrupting adhesions rather than pharmacologically suppressing neural transmission. This distinction is critical: while lidocaine blocks carry 29% to 58% false-negative rates [13,50], our patient demonstrated immediate functional improvement post-hydrodissection despite lacking response to prior physiotherapy and pharmacological interventions.

### 3.3. Mechanistic Advantages of Fascial-Targeted Approach

The role of crural fascia in sural dysfunction provides a therapeutic rationale for fascial release. Psoriasis-induced inflammation may increase fascial stiffness through collagen cross-linking [51], creating a dynamic compartment syndrome during ankle motion. Hydrodissection directly addresses this by mechanically separating fascial–neural adhesions, restoring physiologic nerve gliding, and disrupting inflammatory cascades perpetuating fibrosis.

The 50 cc D5W volume provides sufficient hydraulic pressure to dissect fascial planes while avoiding neural toxicity concerns associated with local anesthetics. The sustained 18-month relief observed suggests this approach may modify disease progression rather than merely suppressing symptoms—an important advantage over transient pharmacologic interventions.

The targeted delivery of the injectate, facilitated by real-time ultrasound guidance, allowed for precise circumferential hydrodissection of the SN, ensuring complete release from surrounding tissues. The use of SDP was instrumental in both diagnosing and confirming the SN involvement, enabling precise localization of the point of maximal restriction. The correlation between functional impairment demonstrated by SDP and morphological evidence of nerve swelling provides robust validation of our diagnostic approach. This combined methodology offers a more comprehensive evaluation compared to static imaging alone, allowing the clinician to identify subtle nerve pathology that might be overlooked in standard ultrasound examination. The immediate post-hydrodissection SDP findings, which demonstrated improved nerve mobility and absence of pain reproduction, further validated the effectiveness of the intervention.

Notably, no studies have specifically investigated US-guided HD for sural nerve dysfunction in psoriasis patients. However, psoriasis, an immune-mediated inflammatory disease with systemic manifestations [1,2], may predispose individuals to nerve dysfunction via multiple pathways:

- **Inflammation of surrounding tissues:** Pro-inflammatory cytokines (e.g., TNF-alpha [52,53]) cause swelling/thickening in muscles, tendons, and fascia, potentially compressing adjacent nerves (e.g., crural fascia inflammation compressing the sural nerve) [54].
- **Fascial changes:** Chronic inflammation induces fascial fibrosis and reduced mobility, restricting nerve gliding and increasing entrapment susceptibility [55].
- **Enthesitis and joint involvement:** Psoriatic arthritis (affecting ~30% of patients [51,56,57,58,59] triggers entheseal/joint inflammation [1,60,61,62], leading to periarticular swelling that may compress peripheral nerves.
- **Neurogenic inflammation:** Sensory nerve fiber proliferation and neurogenic inflammation create a feedback loop exacerbating skin and nerve inflammation [1,59,60].
- **Proximity of inflamed structures:** Inflammation spreads readily to adjacent tissues, directly irritating or compressing nearby nerves [60,62].

**The crural fascia’s role in sural nerve pathology is supported by converging evidence:**

Cadaveric studies and case reports have documented fibrous bands within this fascia compressing the sural nerve [4], while ultrasound biomarkers in this case revealed pathological thickening (>1 mm) and fibrotic tethering [63]. Biomechanically, psoriasis-induced inflammation reduces fascial elasticity through collagen cross-linking [27,28,29,51], creating elevated pressure on nerves during movement [62]. This ‘dynamic compartment syndrome’ mechanism was functionally validated through SDP localization of adhesion-related pain [32,33,34].

In summary, psoriasis-related inflammation can compress peripheral nerves or restrict their mobility, establishing psoriasis as a potential contributor to nerve dysfunction. In our patient, the presence of a psoriatic skin lesion proximal to the nerve involvement site emphasizes a possible association between psoriasis-related inflammation and nerve pathology. Although no overt inflammatory signs were evident on Doppler ultrasound, the nerve dysfunction located beneath the psoriatic skin lesion suggests potential biochemical contributions alongside mechanical factors. Hydrodissection directly targets the fascia surrounding the nerve, mechanically separating the nerve from these compressive forces. The injected fluid not only creates space but may also help to break down adhesions and improve fascial mobility.

### 3.4. The Koebner Phenomenon and Potential Neural Involvement

The anatomical contiguity of the psoriatic plaque with the site of sural nerve dysfunction raises an intriguing conceptual possibility regarding the Koebner phenomenon—a well-documented feature of psoriasis in which new skin lesions develop at sites of trauma or irritation. While the Koebner phenomenon is classically described as a dermatological process, recent evidence suggests that the inflammatory responses underlying this phenomenon may not be strictly limited to dermal structures. In our patient, the close spatial relationship between the psoriatic lesion and the nerve pathology suggests that local inflammatory cascades—potentially including those involved in Koebner-like phenomena—may extend beyond the dermis to affect adjacent neural tissues. Pro-inflammatory cytokines such as TNF-α and IL-17, which are elevated in psoriatic lesions and play central roles in psoriatic inflammation [52,53], are known to directly affect nerve tissues and can contribute to neurogenic inflammation and sensitization [59,60]. We hypothesize that in this patient, the localized psoriatic inflammation at the calf lesion—potentially representing an extension or manifestation of Koebner-like inflammatory processes—may have triggered or exacerbated fascial fibrosis and nerve compression at the anatomically proximal sural nerve. However, it is important to emphasize that this proposed link between Koebner-like phenomena and neural involvement remains speculative based on this single case. The exact molecular and cellular mechanisms by which localized psoriatic inflammation might affect adjacent nerve tissues are not yet elucidated and would require formal investigation through mechanistic studies.

## 4. Limitations

This study has several important limitations that should be acknowledged.

First, it represents a single case report, and therefore the proposed association between localized psoriatic inflammation and sural nerve dysfunction should be interpreted as exploratory and hypothesis-generating rather than conclusive [51,59]. The mechanistic link between focal inflammation and nerve compression remains speculative, and potential coincidental coexistence cannot be entirely excluded.

Second, several methodological limitations exist. Electrodiagnostic (EDX) confirmation and serial ultrasonography were not performed, which restricts the objective assessment of neural recovery and morphological evolution [9,10,11,12]. Although the decision to omit EDX was based on the patient’s acute clinical presentation and its limited diagnostic sensitivity for fascial-restriction neuropathies, future studies should include standardized neurophysiologic recordings and quantitative bilateral nerve mapping. Similarly, the absence of inflammatory biomarker evaluation (e.g., TNF-α, IL-6, IL-17) limits verification of the proposed local immunologic contribution [52,53,60].

Third, operator-dependent variability in advanced ultrasonographic techniques such as sonoguided digital palpation (SDP) and hydrodissection should be recognized. This method requires specialized training and may limit reproducibility across practitioners [3,25].

Finally, the generalizability of these findings to broader psoriasis populations remains uncertain. Larger case series incorporating objective functional outcomes, longer follow-up, and histopathologic or biomarker correlation are needed to substantiate these preliminary observations [1,14,15,16].

In conclusion, while this report highlights a potentially novel hypothesis regarding inflammation-induced fascial–neural entrapment in psoriasis, the findings should be interpreted with caution. Future prospective, multimodal investigations are necessary to clarify causal mechanisms and validate the therapeutic role of hydrodissection in psoriatic neuropathic pain [17,24,27].

## 5. Conclusions

In summary, while this case provides valuable preliminary evidence for a novel diagnostic and therapeutic approach to sural nerve dysfunction in a psoriasis patient, the findings must be interpreted with appropriate caution due to the single-case design, absence of electrodiagnostic and biomarker confirmation, limited functional outcome measures, and the speculative nature of the proposed mechanistic pathways. Future prospective studies incorporating comprehensive diagnostic protocols, objective outcome measures, biomarker analysis, and larger patient cohorts are essential to validate these findings and establish evidence-based guidelines for managing peripheral neuropathies in psoriasis patients.

## Data Availability

The original contributions presented in this study are included in the article/Supplementary Materials. Further inquiries can be directed to the corresponding authors.

## Supplementary Materials

Video S1. Real-time ultrasound imaging during Sonoguided Digital Palpation (SDP) of the abnormal sural nerve (SN). This video demonstrates dynamic assessment of the SN in the symptomatic right calf of a patient with psoriasis and heel pain. The examination reveals sonographic features consistent with SN dysfunction due to fascial restriction. Note the fatty triangle within the crural fascia housing the SN and lesser saphenous vein (LSV). Key findings include: (1) increased hyperechoic fibrotic tissue surrounding the SN, (2) significantly restricted nerve mobility (<5 mm transverse excursion), and (3) anechoic signals suggesting perineural edema. The video correlates palpating finger movements with real-time ultrasound imaging, demonstrating SDP’s ability to localize the point of maximal restriction and reproducibly elicit the patient’s concordant heel pain, confirming functional impairment. https://www.dropbox.com/scl/fi/9n8w520bvag1r6nwlafot/SDP-Sural-Nerve-Figured_Abnormal.mp4?rlkey=hdt8801bcuslf4fgauwlay3pp&dl=0 (accessed on 22 October 2025); Video S2. Real-time ultrasound imaging during Sonoguided Digital Palpation (SDP) of the contralateral (asymptomatic) sural nerve (SN). This video demonstrates the dynamic assessment of the normal SN in the left calf of the patient, serving as an internal control for comparison with the affected right side. The examination reveals normal gliding mobility of the SN (as indicated by the yellow arrow) within the fatty triangle of the crural fascia, which houses the SN and lesser saphenous vein (LSV). Key findings include: physiological gliding mobility of the SN exceeding 5 mm transverse excursion and absence of perineural edema or fibrotic tissue changes. This contralateral comparison quantitatively demonstrates the restricted mobility observed on the symptomatic side. https://www.dropbox.com/scl/fi/fbpdcd9tnsb1kpyoy569n/SDP-Sural-Nerve-Figured_normal.mp4?rlkey=ja9tg6snw8szvm5d8jnl3p2rh&dl=0 (accessed on 22 October 2025); Video S3. Ultrasound-guided hydrodissection of the sural nerve. The video demonstrates the in-plane approach for hydrodissection, with the hyperechoic needle (arrow) advancing towards the sural nerve (SN). The injectate (5% dextrose in water, D5W) is seen expanding circumferentially around the SN, achieving mechanical release from surrounding adhesions. Anatomical landmarks are labeled: MG—medial gastrocnemius, LG—lateral gastrocnemius, FHL—flexor hallucis longus, TN—tibial nerve. The video captures the immediate restoration of nerve mobility following hydrodissection, showcasing the separation of the SN from constrictive fibrotic tissue and the subsequent improvement in gliding dynamics. https://www.dropbox.com/scl/fi/x1vdnuus5vb2vzj0uc7w5/SN-HD-with-explanation-and-labeled.mp4?rlkey=ns1oyy05pdei5lyoaxfol3a0a&dl=0 (accessed on 22 October 2025).
